# Assessment of Treatment Response in Patients With Diffuse Large B-cell Non-Hodgkin Lymphoma Using the Deauville Criteria: A Case Report and Literature Review

**DOI:** 10.7759/cureus.89983

**Published:** 2025-08-13

**Authors:** David Gutierrez Albenda, Juan José Solano-Brenes, Nelson Mauricio Sánchez Hidalgo, Laura Natalia Rodríguez Varela, Paula Ulate Blanco

**Affiliations:** 1 Cyclotron-PET/CT Laboratory, University of Costa Rica, San José, CRI; 2 School of Medicine, University of Costa Rica, San José, CRI

**Keywords:** 18f-fluorodeoxyglucose positron emission tomography (18f-fdg pet), case report, deauville score, end-of-treatment response, large b-cell lymphoma, r-chop therapy

## Abstract

Diffuse large B-cell lymphoma (DLBCL) represents the most prevalent subtype of non-Hodgkin lymphoma in the adult population and is typically associated with an aggressive clinical trajectory. Despite significant therapeutic advancements, a proportion of patients exhibit resistance to first-line chemotherapy, underscoring the need for accurate and early assessment of treatment response to inform prognosis and optimize therapeutic strategies. We describe the case of a 64-year-old male with a medical history notable for ischemic heart disease and metabolic syndrome who presented with abdominal pain and unintentional weight loss. Diagnostic evaluation confirmed a diagnosis of DLBCL, with initial F-fluorodeoxyglucose positron emission tomography/computed tomography (FDG-PET/CT), revealing extensive nodal and extranodal disease involvement. Following the completion of six cycles of frontline chemotherapy, end-of-treatment FDG-PET/CT demonstrated substantial resolution of metabolic activity, corresponding to a Deauville score of 2, consistent with a complete metabolic response. The Deauville five-point scoring system is a standardized method for interpreting PET/CT scans in lymphoma, comparing lesion uptake to the mediastinum and liver to assess treatment response. Consequently, no additional consolidation therapy was pursued. This case illustrates the pivotal role of FDG-PET/CT, interpreted using the Deauville five-point scale, as a reliable and clinically relevant modality for evaluating treatment response in DLBCL, thereby facilitating informed decision-making regarding the continuation or cessation of therapy.

## Introduction

Diffuse large B-cell lymphoma (DLBCL) represents the most common non-Hodgkin lymphoma (NHL) in adults, accounting for approximately 30-40% of newly diagnosed NHL cases [1]. This lymphoma subtype is characterized by an aggressive course. Despite advances in research and treatment, cure rates remain suboptimal, with approximately one-third of patients failing to achieve long-term remission following first-line therapy with six cycles of R-CHOP, a regimen that includes cyclophosphamide, doxorubicin, vincristine, prednisone, and monoclonal anti-CD20 antibody rituximab [2]. Approximately 65% of patients achieve and sustain complete remission following first-line therapy. However, 10-15% have treatment-refractory disease, while 20-25% experience relapse after an initial response [2,3].

DLBCL displays marked heterogeneity due to underlying genetic and immunophenotypic variations, which contribute to the variability in prognosis and therapeutic outcomes. Therefore, accurate and early assessments of treatment responses are critical for predicting prognosis and guiding clinical decision-making to improve cervical rates [3].

F-fluorodeoxyglucose positron emission tomography/computed tomography (FDG-PET/CT) has emerged as a valuable imaging modality for staging, treatment monitoring, and prognostication in hematologic malignancies [3,4]. FDG is a glucose analog labeled with fluorine-18 (¹⁸F), a positron-emitting radioisotope. It accumulates in cells with high glycolytic activity, such as malignant lymphocytes, but cannot undergo further metabolism, becoming trapped intracellularly. As fluorine-18 decays, it emits a positron that travels a short distance before interacting with an electron, resulting in the conversion of their combined mass into two gamma photons emitted in opposite directions. PET scanners detect these coincident photons and reconstruct a three-dimensional map of radiotracer distribution, reflecting metabolic activity. CT provides high-resolution anatomical localization and enables attenuation correction, improving the accuracy and specificity of PET findings [3].

Compared to conventional CT, FDG PET/CT allows for the distinction between viable tumor tissue and post-treatment fibrosis or necrosis, thereby reducing unnecessary treatment modifications or invasive procedures [3]. Sensitivity of CT at the end of treatment was 57.5%, specificity was 86.7%, and accuracy was 71.6%, as compared to the 100% sensitivity and specificity of PET/CT [5]. However, traditional metabolic PET/CT parameters such as maximum standardized uptake value (SUVmax), metabolic tumor volume, and total lesion glycolysis (TLG) have limited capacity to fully capture tumor heterogeneity [6]. 

The Deauville five-point scale, introduced in 2009, provides a simple and reproducible method for evaluating early metabolic response to immunochemotherapy in lymphoma patients. This scoring system compares FDG uptake in residual lesions to uptake in the liver and mediastinal blood pool, facilitating objective response assessment. Studies have demonstrated high accuracy and inter-observer agreement with the Deauville criteria in lymphoma response evaluation [7,8].

This report presents a case of DLBCL and highlights the utility of FDG-PET/CT and the Deauville scale in assessing response to R-CHOP therapy. 

## Case presentation

A 64-year-old male with a medical history of ischemic heart disease, metabolic syndrome, mild antral erythematous gastritis, and a 20-year history of smoking (now ceased) presented with a one-year history of abdominal pain localized to the epigastric and right upper quadrant regions, accompanied by unintentional weight loss. He denied systemic symptoms such as fever or night sweats. On physical examination, bilateral cervical lymphadenopathy was noted.

Initial diagnostic workup included an upper gastrointestinal endoscopy, which revealed an H1 duodenal ulcer with features suspicious for dysplasia. Abdominal ultrasound demonstrated left calyceal ectasia, without other notable abnormalities. 

Histopathological examination of duodenal bulb biopsy specimens revealed features consistent with large B-cell lymphoma, not otherwise specified (NOS). Bone marrow aspiration and biopsy showed no evidence of lymphoproliferative infiltration.

**Figure 1:**
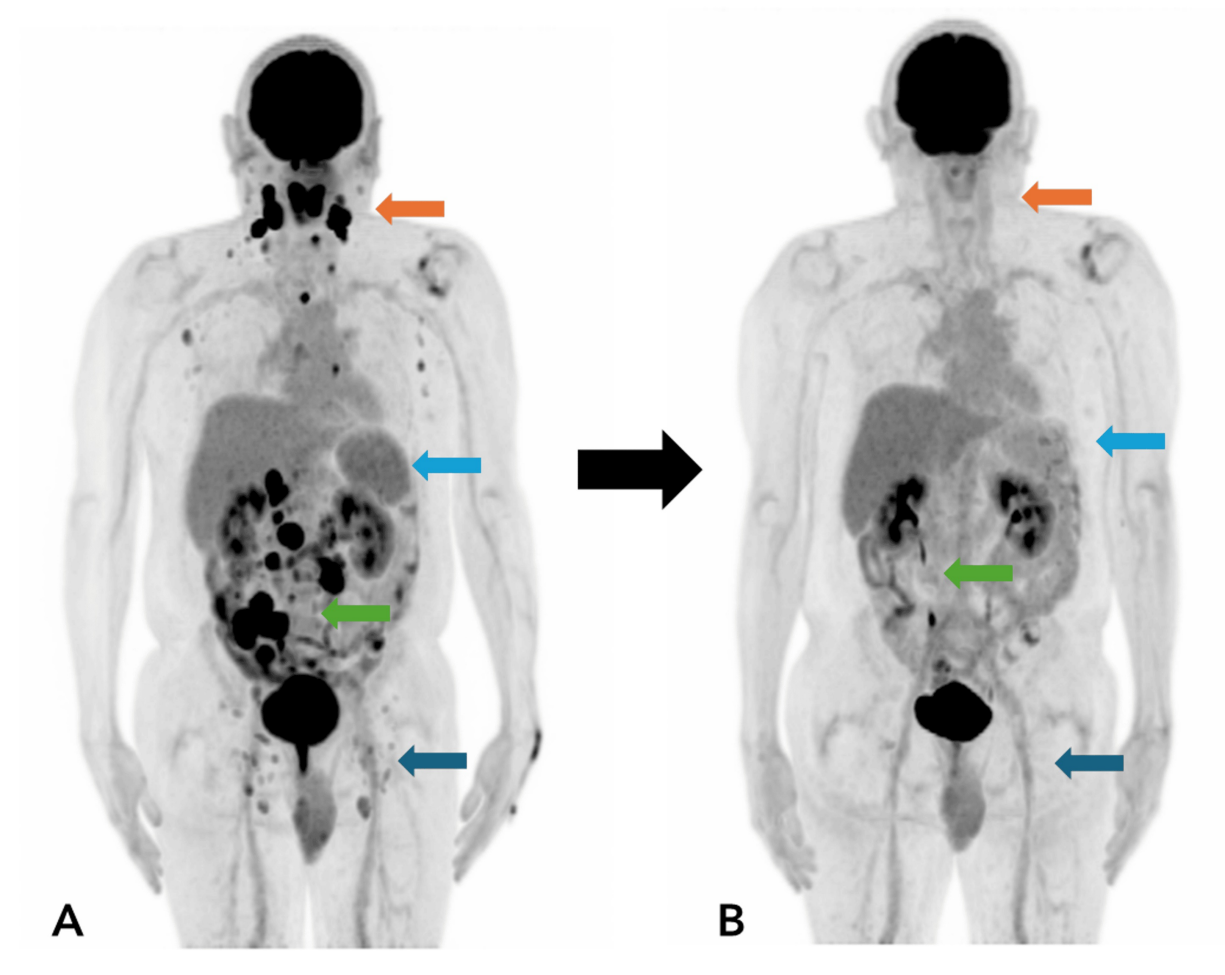
a) The maximum intensity projection (MIP) image from the FDG-PET/CT performed in November 2024 demonstrates intense hypermetabolic activity involving both the supradiaphragmatic and infradiaphragmatic lymph node regions, with additional extranodal involvement of osseous structures and intestinal segments. FDG-avid involvement of Waldeyer’s ring is indicated by the orange arrow. A reversal of the normal liver-to-spleen metabolic gradient is observed (light blue arrow). Asymmetric hypermetabolic thickening of the first portion of the duodenum and terminal ileum is noted (green arrow). Bilateral iliac chain and inguinal lymphadenopathy with increased FDG uptake is also evident (dark blue arrow). b) Follow-up MIP image from the FDG-PET/CT performed in May 2025, following completion of six cycles of R-CHOP chemotherapy, demonstrates complete or near-complete metabolic resolution of previously noted nodal and extranodal lesions. Residual uptake is below hepatic background levels. Arrows correspond to sites of prior disease involvement, now showing metabolic response consistent with the treatment effect.

Baseline FDG-PET/CT demonstrated intense metabolic activity in multiple nodal and extranodal regions, as shown in Figure 1a. Reference background SUVmax were 2.5 in the liver and 2.0 in the mediastinal blood pool. Cervical lymphadenopathy extended from levels I to V, with the most significant uptake in the right level IIa lymph node (SUVmax: 27.5). Additional hypermetabolic lymph nodes were present in bilateral superior/inferior paratracheal chains, the subcarinal area, and the left perihilar region (SUVmax: 5.0, left perihilar). Bilateral axillary involvement was observed, with peak uptake on the left (SUVmax: 3.8).

Abdominal and pelvic PET/CT revealed widespread hypermetabolic lymphadenopathy involving the para-aortic, interaortocaval, precaval, retrocaval, and bilateral iliac chains (SUVmax: 25.8, interaortocaval region), as well as bilateral inguinal nodes (SUVmax: 4.0, right side). Numerous metabolically active mesenteric nodules were also present. 

**Figure 2:**
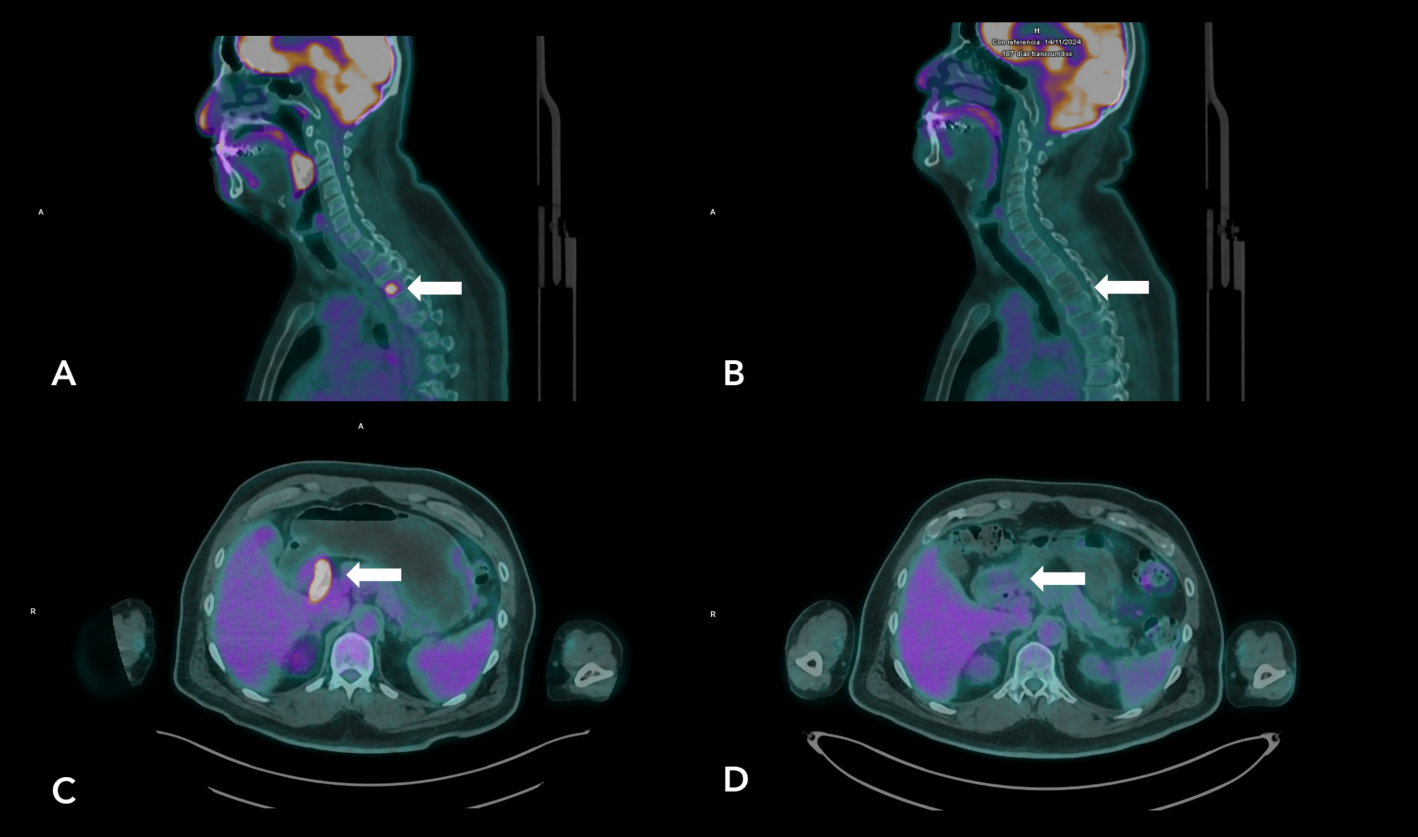
a, b) Sagittal PET/CT fusion images. At staging (a), a hypermetabolic lesion is observed in the T3 vertebral body, with resolution of hypermetabolism following six cycles of R-CHOP (b); the area now demonstrates hypometabolism. c, d) Axial PET/CT fusion images. At staging (c), a hypermetabolic lesion is identified in the first portion of the duodenum, with resolution of hypermetabolism after six cycles of R-CHOP (d); the activity is now within physiological levels.

Involvement of Waldeyer’s ring was evident, particularly the left palatine tonsil, which measured up to 2 cm with intense uptake (SUVmax: 19.3). A reversal of the physiological liver-spleen metabolic gradient was noted, with a splenic SUVmax of 3.2. Extranodal disease included a focal lesion in the T3 vertebral body (SUVmax 10.4) and asymmetric thickening of the first portion of the duodenum (SUVmax 16.9) (Figure 2a, 2c). The terminal ileum demonstrated the most intense activity (SUVmax: 20.2), with associated fat stranding and obliteration of adjacent planes. 

Following six cycles of R-CHOP chemotherapy, an end-of-treatment FDG PET/CT scan was performed. The study demonstrated marked resolution of previously identified hypermetabolic lymphadenopathy as shown in Figure 1b. In the cervical region, only a few small residual nodes remained, the largest measuring 0.8 cm at the short axis (SUVmax: 1.2, level IIa). In the thorax, residual paratracheal nodes were non-pathological. In the abdomen and pelvis, small residual retroperitoneal lymph nodes remained (largest 1 cm, SUVmax: 1.6), without pathological uptake. Mesenteric lymphadenopathy was significantly reduced in number and size, and all were metabolically inactive. 

In Figure 2b and Figure 2d, complete resolution of hypermetabolic activity was observed in the Waldeyer's ring, the first portion of duodenum, the terminal ileum, and the focal lesion in the T3 vertebral body. In addition, the splenic metabolic activity returned to physiological levels, and the spleen's morphology and dimensions were preserved.

## Discussion

The FDG PET/CT has become the gold standard for staging and evaluating treatment response in patients with diffuse large B-cell (DLBCL), owing to its ability to detect changes in tumor metabolic activity earlier than anatomical changes detected by conventional imaging [3,9].

Numerous studies support the use of FDG PET/CT at baseline, during interim assessments, and at the end of the treatment. These scans help clinicians better stratify risks, assess therapeutic response, and guide post-treatment management with the ultimate goal of improving remission and survival outcome [3].

In patients with DLBCL, persistent metabolic activity on end-of-treatment PET/CT after six cycles of R-CHOP is considered a marker of poor prognosis, irrespective of disease stage. In fact, PET/CT-positive patients have demonstrated an event-free survival rate of approximately 48%, compared with 78% for PET/CT-negative patients [6,10]. 

End-of-treatment PET/CT demonstrating a complete response holds significant prognostic value for both progression-free survival and overall survival in patients with DLBCL following immunochemotherapy. Studies have shown a five-year overall survival rate of approximately 75% in patients achieving a complete metabolic response on PET/CT, compared to only 35% in those without a complete response [11].

To standardize response interpretation, the Deauville five-point scale was introduced. This semi-quantitative visual scoring system evaluates residual FDG uptake in comparison to physiological uptake in the mediastinum and liver [3,7]. Its simplicity and reproducibility have made it a preferred tool in clinical practice and trials for assessing treatment response in FDG-avid lymphoma

Based on the case presented above and Table 1, the patient achieved a Deauville score of 2 at the end of six R-CHOP cycles, indicating a complete metabolic response. The absence of residual FDG-avid lesions with uptake below liver background supports treatment success and predicts a favorable prognosis. As a result, further consolidative radiotherapy was not indicated, aligning with evidence that suggests PET-negative patients can safely forgo additional irradiation [10,12].

**Table 1:** Deauville scale for therapy stratification in patients with F-fluorodeoxyglucose (FDG)-avid lymphomas. Source: [6]

Score	Criteria	Interpretation
1	No FDG uptake	Complete metabolic response
2	FDG uptake lower than or equal to the mediastinal blood pool	
3	FDG uptake higher than the mediastinal blood pool but lower or equal to the liver	
4	FDG uptake moderately increased compared to the liver	Partial metabolic response; stable disease; progressive disease
5	FDG uptake markedly increased compared to the liver and/or new sites of disease	

Numerous international studies and meta-analyses affirm that end-of-treatment PET/CT, when interpreted using the Deauville criteria, is predictive of clinical outcomes. The scoring system has demonstrated a high negative predictive value (NPV ~85%), confirming its reliability in identifying patients who are unlikely to relapse. The GOYA trial further supported these findings, demonstrating that patients with a complete metabolic response (Deauville score 1-3) on end-of-treatment PET/CT had significantly better progression-free survival (PFS) and overall survival (OS) [13].

In cases with a Deauville score of 4 or 5 (a positive end-of-treatment PET/CT), serial imaging is recommended to confirm or exclude residual disease. A biopsy should be considered for lesions with a Deauville score of 5. Among patients with this score, 50-75% experience disease progression, with a reported five-year time to progression (TTP) of approximately 33%. In contrast, patients with a Deauville score of 4 have more favorable outcomes, with a five-year TTP of 87%, closely aligning with those who scored 1-3 (five-year TTP of 91%) [10,13].

Patients with negative end-of-treatment PET/CT findings were typically managed with observation, regardless of initial tumor bulk. Conversely, those with a Deauville score of 4 or 5 were often offered consolidation radiotherapy. Evidence suggests that this approach may help mitigate the inferior prognosis associated with a positive PET/CT scan [10,13,14].

However, limitations of the Deauville criteria have been reported. For instance, the positive predictive value (PPV) remains moderate (~44%), indicating a considerable risk of false-positive results. This limitation may lead to the misclassification of patients as having residual disease, potentially resulting in overtreatment. Therefore, additional clinical or pathological evaluation is often warranted in cases with Deauville scores of 4-5. Other limitations include inter-observer variability, the subjectivity of visual interpretation, and the inability to reliably distinguish between residual disease and post-treatment inflammation [4,13,15].

Overall, the five-point Deauville scale remains a cornerstone in the evaluation of metabolic response in DLBCL due to its simplicity, accessibility, and clinical relevance. When applied in conjunction with clinical judgment, it supports more accurate patient management and may help avoid unnecessary interventions.

## Conclusions

FDG-PET/CT, interpreted using the Deauville five-point scale, is a valuable tool for assessing treatment response in DLBCL. In this case, a Deauville score of 2, achieved after six cycles of R-CHOP immunochemotherapy, indicated a complete metabolic response and suggested that consolidative radiation therapy is not required. The Deauville scores offer a practical and reproducible method for evaluating early treatment response, guiding clinical decision-making, and predicting favorable long-term outcomes in DLBCL patients. Continued research and standardization of FDG-PET/CT use are crucial to optimize its role in lymphoma management.
